# *Borrelia persica* Infection in Immunocompetent Mice - A New Tool to Study the Infection Kinetics In Vivo

**DOI:** 10.1371/journal.pntd.0004404

**Published:** 2016-02-18

**Authors:** Sandra Schwarzer, Evelyn Overzier, Walter Hermanns, Gad Baneth, Reinhard K. Straubinger

**Affiliations:** 1 Bacteriology and Mycology, Institute for Infectious Diseases and Zoonoses, Ludwig-Maximilians-Universität München, Munich, Germany; 2 Institute of Veterinary Pathology, Ludwig-Maximilians-Universität München, Munich, Germany; 3 Koret School of Veterinary Medicine, Hebrew University of Jerusalem, Rehovot, Israel; University of Connecticut Health Center, UNITED STATES

## Abstract

*Borrelia persica*, a bacterium transmitted by the soft tick *Ornithodoros tholozani*, causes tick-borne relapsing fever in humans in the Middle East, Central Asia and the Indian peninsula. Immunocompetent C3H/HeOuJ mice were infected intradermally with *B*. *persica* at varying doses: 1 x 10^6^, 1 x 10^4^, 1 x 10^2^ and 4 x 10^0^ spirochetes/mouse. Subsequently, blood samples were collected and screened for the presence of *B*. *persica* DNA. Spirochetes were detected in all mice infected with 1 x 10^6^, 1 x 10^4^ and 1 x 10^2^ borrelia by real-time PCR targeting the *flaB* gene of the bacterium. Spirochetemia developed with a one- to two-day delay when 1 x 10^4^ and 1 x 10^2^ borrelia were inoculated. Mice injected with only four organisms were negative in all tests. No clinical signs were observed when infected mice were compared to negative control animals. Organs (heart, spleen, urinary bladder, tarsal joint, skin and brain) were tested for *B*. *persica*-specific DNA and cultured for the detection of viable spirochetes. Compiled data show that the target organs of *B*. *persica* infections are the brain and the skin. A newly developed serological two-tiered test system (ELISA and western blot) for the detection of murine IgM, IgG and IgA antibody titers against *B*. *persica* showed a vigorous antibody response of the mice during infection. In conclusion, the infection model described here for *B*. *persica* is a platform for in vivo studies to decipher the so far unexplored survival strategies of this *Borrelia* species.

## Author Summary

The spirochete *Borrelia persica* is a tick-borne bacterium that is transmitted by the vector *Ornithodoros tholozani* to its vertebrate host in the Middle East, Central Asia and the Indian peninsula. Current migration of vast numbers of individuals from this area increases the likelihood that *B*. *persica* infections will be introduced into new geographic regions. After infection and distribution by the bloodstream, relapsing fever episodes occur in humans. Since no reliable in vivo tools have existed so far to study this organism, a murine model was established in this study to characterize the infection kinetics in immunocompetent mice. Aspects of the potential infectivity of the laboratory strain and of potential clinical signs, spirochetemia and antibody response as well as organ tropism and histopathological reactions were studied. With the successful infection model presented here, further studies are conceivable in order to gain advanced insights into the pathogenesis of *B*. *persica* infection and to characterize in detail the host immune response mounted against the bacterium. We propose that this model could also be used for the development of new rapid diagnostic approaches to initiate or monitor treatment regimes in order to clear or prevent the infection with *B*. *persica*.

## Introduction

Spirochetes of the genus *Borrelia* (*B*.) are vector-borne, spiral-shaped bacteria that can be divided into two functional groups [1]. One large group of spirochetes belongs to the *B*. *burgdorferi* sensu lato complex (e.g. *B*. *burgdorferi* sensu stricto, *B*. *afzelii*, *B*. *garinii*, *B*. *bavariensis*). Lyme disease borreliae are transmitted by hard-shelled *Ixodes* ticks [2]. The second group includes the relapsing fever (RF) borreliae which spread primarily via soft ticks with an exception of *B*. *recurrentis* that is transmitted by the body louse (*Pediculus humanus*). Among others, *B*. *hermsii*, *B*. *duttonii* and also *B*. *persica* are tick-borne RF borreliae which induce tick-borne relapsing fever (TBRF) (reviewed in [3]). *B*. *persica* is transmitted by the soft tick *Ornithodoros tholozani* during blood meals [4]. These ticks are prevalent in areas such as the Middle East, Central Asia and the Indian peninsula and feed on humans as well as on animals (reviewed in [5]). Moreover, TBRF can occur in non-endemic countries due to travel of infected people [6, 7]. Clinically, the disease manifests with fever attacks in human patients related to high numbers of spirochetes in the blood circulation during fever episodes [8–10] and non-specific clinical signs such as chills, headache, nausea, vomiting, sweating, abdominal pain, arthralgia, cough and photophobia which may occur [9]. Rodhain reviewed as early as 1976 [11] that a high level of experimental pathogenicity of *B*. *persica* can be perceived in guinea pigs, hedgehogs and rabbits whereas lower levels seem to occur in monkeys, adult white mice and rats. So far primarily guinea pigs have been used to multiply *B*. *persica* [12–14] and just recently it was possible to maintain *B*. *persica* in vitro [14, 15]. However, for other RF borrelia species the mouse is usually considered to be the appropriate animal model [16–19] and Babudieri investigated relapsing fever in Jordan by injecting blood of diseased patients into mice in order to confirm TBRF spirochetosis [20]. The mice used in the experiment tested positive two to five days after injection. Furthermore, Babudieri studied the infection rate of captured *Ornithodoros tholozani* ticks. Squashed ticks were inoculated into mice, but infection was not initiated, while a very low infection rate was obtained when the ticks were allowed to feed directly on these animals. Spirochetes were not present constantly and uniformly in the mice’s blood. In addition, the author mentioned that the spirochetes survived in the mice’s brains. In 2006, Assous et al. inoculated intraperitoneally blood of TBRF patients from Israel into ICR mice and detected spirochetes in the mice’s blood samples on day four as well as on day six of the experiment [21]. Since comprehensive data for *B*. *persica* in mice were not available, we aimed to establish and characterize in detail an infection model for *B*. *persica* in immunocompetent mice. Therefore, we infected intradermally 44 C3H/HeOuJ mice with *B*. *persica* strain LMU-C01. In order to gain insight into the infection with these TBRF spirochetes, we investigated (a) whether this laboratory strain of *B*. *persica* is able to establish an infection in immunocompetent mice; (b) whether the mice develop clinical signs; (c) when and in which quantity the spirochetes appear in the blood circulation; (d) confirm that *B*. *persica* disseminates into organs and investigate the histopathological changes; (e) characterize the mice’s immune response during infection; and (f) define the minimal dose necessary to infect animals. After compilation of all data, we came to the conclusion that the infection model described here is a reliable tool that can be used for further research studies.

## Materials and Methods

### *B*. *persica* in vitro cultivation

For this study, 100 μl of thawed *B*. *persica* passages (strain LMU-C01, isolated from a cat in Israel; passage 2, 3.9 x 10^6^ organisms/ml) were cultivated in Pettenkofer/LMU Bp medium as described previously [15]. Cultures were incubated for five days and viable bacteria were counted with a Petroff-Hausser counting chamber (Hausser Scientific, Horsham, Pennsylvania, USA). Bacteria suspensions were adjusted to the required cell concentration by dilution of cultures with plain medium.

### Mice and intradermal injection of *B*. *persica*

In total, 54 six- to seven-week-old female C3H/HeOuJ mice (Charles River Wiga Deutschland GmbH, Sulzfeld, Germany) were kept in individually ventilated cages (ISOcage N System; Tecniplast Deutschland GmbH, Hohenpeißenberg, Germany) at the animal facility of the Institute for Infectious Diseases and Zoonoses, Ludwig-Maximilians-Universität (Munich, Germany). Animals were manipulated in laminar flow systems in order to sustain specific-pathogen-free conditions. The health status of all mice and the body temperature, which was measured with a subcutaneous transponder (IPTT-300 Temperature Transponders; Plexx B. V., PW Elst, Netherlands), were recorded twice a day.

Initially, 20 mice were exposed to 1 x 10^6^ *B*. *persica* spirochetes in 100 μl medium by intradermal injection into the shaven back. In addition, four animals were injected with 100 μl medium alone and served as negative controls. The injection volume was divided into small portions (10 x 10 μl), placed close to each other into the skin (~ 4 cm^2^ area). For the dose finding study, eight mice per group were injected with *B*. *persica* suspensions with varying concentrations. Group #1: 1 x 10^4^ spirochetes per mouse; group #2: 1 x 10^2^ spirochetes per mouse; group #3: 4 x 10^0^ spirochetes per mouse. Two additional animals in each group served as negative and infection/transmission controls.

### Blood, plasma and serum samples

To study the kinetics of bacteremia and the development of specific antibodies post infection, blood samples were collected at preassigned intervals. Two drops of blood were collected in a Microvette 100 K3E (preparation K_3_EDTA; Sarstedt AG & Co., Nümbrecht, Germany) by facial bleeding after cutting the skin with a 4-mm Goldenrod Animal Lancet (Braintree Scientific, BioMedical Instruments, Zöllnitz, Germany). The bleeding scheme was as follows: during the first two weeks each mouse was bled every second day. However, in order to collect data for each single day of the first 14-day interval, the group was divided into two equal subgroups and these subgroups were bled according to alternating schedules. After day 14, all animals were bled together once a week until the final days 49/50. The bleeding scheme for the dose finding study was: during the first 20 days, each animal was bled every second day. Subgroups were formed and bled according to alternating schedules to obtain blood samples for each experimental day. After day 20, blood samples were collected every second day up to the final days 30/31/32. Alternating schedules were applied to the subgroups (each mouse was bled every fourth day).

For DNA-extraction, 5 μl from each blood sample were transferred into a 1.5-ml safe-lock tube (Eppendorf Vertrieb Deutschland GmbH, Wesseling-Berzdorf, Germany) and frozen at -30°C until used. Surplus blood samples of the regular blood collection from animals that had received 1 x 10^6^ *B*. *persica* organisms were pooled subgroup-specific in another 1.5-ml safe-lock tube for plasma production. After euthanasia, a final blood sample of each mouse was collected in a micro tube (1.1ml Z-Gel; Sarstedt AG & Co.) for serum production. Plasma and serum preparation were done by a two-time centrifugation (Centrifuge 5430 R V 1.1, rotor FA-45-30-11; Eppendorf Vertrieb Deutschland GmbH) at 350 x g for 10 min at 24°C. The supernatants were collected in a 1.5-ml safe-lock tube and were frozen at -30°C until serological analyses were performed.

### *B*. *persica* DNA quantification in blood and DNA detection in tissue samples

AS3000 Maxwell 16 MDx Instrument and the Maxwell 16 LEV Blood DNA Kit (Promega GmbH, Mannheim, Germany) were used for DNA extraction from blood and tissue samples. In the case of blood: 5 μl thawed blood, 300 μl sterile phosphate-buffered saline (PBS), 300 μl lysis buffer and 30 μl Proteinase K were mixed. The following steps were done according to the manufacturer’s technical manual # TM333 (Maxwell 16 LEV Blood DNA Kit and Maxwell 16 Buccal Swab LEV DNA Purification Kit Technical Manual; Promega GmbH). DNA was eluted in 60 μl elution buffer and frozen at -30°C. In the case of tissue: 200 μl of incubation buffer (Promega GmbH) were filled in a 1.5-ml safe-lock tube containing thawed tissue (weight less than 50 mg). Then, 200 μl lysis buffer and 30 μl Proteinase K were added and the tissue sample was squeezed and disrupted with a micro pestle (Faust Lab Science GmbH, Klettgau, Germany). Samples were incubated in a ThermoMixer comfort 5355 V 2.0 (Eppendorf Vertrieb Deutschland GmbH) at 56°C and 500 rpm overnight. Additional 200 μl of lysis buffer were added and DNA was extracted with the Maxwell 16 MDx Instrument. DNA was eluted and frozen as written above.

*B*. *persica* DNA was detected with a real-time quantitative PCR (qPCR) assay in a Mx3005P qPCR System (Agilent Technologies Sales & Services GmbH & Co.KG, Böblingen, Germany). The primers and the probe were designed according to the *flaB* target gene of *B*. *persica* using the software Primer3Plus (Free Software Foundation, Inc., Boston, Massachusetts, USA; http://primer3plus.com; [22]). Synthesis of following sequences was carried out by Eurofins Genomics (Ebersberg, Germany): Bp_flaB_fw 5’-GAG GGT GCT CAA CAA GCA A-3’, Bp_flaB_probe 5’-FAM-AAA TCA GGA AGG AGT ACA ACC AGC AGC A-3’-TAM and Bp_flaB_re 5’-CAA CAG CAG TTG TAA CAT TAA CTG G-3’. The expected amplicon size was 106 base pairs. Real-time PCR was carried out in 96 Multiply PCR plate natural (Sarstedt AG & Co.) containing 1.2 μl of each primer (final concentration 600 nM), 0.8 μl of the probe (final concentration 200 nM), 10 μl GoTaq Probe qPCR Master Mix (2 x; Promega GmbH; final concentration 1 x, added CXR reference dye following the manufacturer’s protocol) and 2.5 μl target DNA solution. The reaction volume was 20 μl in total and was pipetted in duplicate for each DNA sample. The amplification program was as follows: initial activation at 95°C for 5 min, 40 cycles of 95°C for 15 s and 60°C for 60 s and a final step at 25°C for 15 s. In each qPCR run a positive control (*B*. *persica* strain LMU-C01, P3), no template control (NTC, 2.5 μl nuclease-free water; Promega GmbH) and samples for calibration of the standard curve (serial dilution of *B*. *persica* DNA, P4) were included. According to the standard curve (considering slope, efficiency and R-squared value), the absolute spirochete number per ml mouse blood was calculated using the MxPro QPCR Software version 4.10 (Agilent Technologies Sales & Services GmbH & Co.KG) based on threshold cycles (C_t_). Graphs were constructed with the OriginPro 9.1 Software (Additive GmbH, Friedrichsdorf, Germany).

As regards tissue, additional to DNA of *B*. *persica flaB* gene mouse-specific glyceraldehyde-3-phosphate dehydrogenase (GAPDH; TaqMan Gene Expression Assay, Mm99999915_g1, VIC dye-labeled MGB probe, 20 x; Applied Biosystems, Thermo Fisher Scientific GmbH, Ulm, Germany) was detected to control the DNA content in the tissue sample. TaqMan Gene Expression Assay was used according to the manufacturer’s recommendations. The reactions mix (total volume 20 μl) contained 10 μl GoTaq Probe qPCR Master Mix (Promega GmbH; final concentration 1 x, added CXR reference dye), 1 μl of the TaqMan Gene Expression Assay (final concentration 1 x) and 2.5 μl DNA solution. PCR conditions were as described above, with the exception of the initial activation step that was separated into two steps: 50°C for 2 min followed by 95°C for 10 min.

### *B*. *persica* re-cultivation from tissue samples

At the end of the infection study with 1 x 10^6^ borrelia per mouse, animals were euthanized at days 49/50 post infection. Tissue processing was carried out as described previously [23]. Heart, spleen, urinary bladder, left tarsal joint, skin from infection areal and brain were collected from each mouse and divided into two parts (brain into three parts). One part was put in a 1.5-ml safe-lock tube and frozen at -30°C for DNA-extraction. The other part was transferred into a second 1.5-ml safe-lock tube filled with 200 μl of Pettenkofer/LMU Bp medium. Subsequently, the tissue was squeezed and disrupted with a micro pestle and the suspension was transferred into a 12-ml tube (Centrifuge Tube 12; TPP, Faust Lab Science GmbH) filled with 10 ml Pettenkofer/LMU Bp medium. The organ cultures were incubated at 37°C in humidified air for three weeks. Observation for viable mobile spirochetes was performed weekly using a dark-field microscope. The third part of the brain, right kidney and right tarsal joint were transferred into a 50-ml centrifuge tube (114x28mm, PP; Sarstedt AG & Co.) filled with 20 ml of 4% formalin and were stored at room temperature until histopathology analyses were carried out. For the dose finding study the skin, brain, right kidney and right tarsal joint were collected on final days 30/31/32 and prepared as described above.

### Histopathology

Brain parts, right kidneys and right tarsal joints (in 4% formalin) from five infected mice (infection dose 1 x 10^6^ *B*. *persica*/mouse) and one control animal were used for the histopathological evaluations. Parts of the brains and kidneys were embedded in paraffin and were cut into 2–3 μl thin slices. The other parts of brains and kidneys as well as tarsal joints were embedded in plastic and sectioned into 1 μl thin slices. After staining with hematoxylin and eosin (HE) as well as Giemsa, observations for histopathological changes were carried out under a bright-field microscope.

### Antigen production and preparation for antibody detection

A low-passaged culture of *B*. *persica* (strain LMU-C01) was used for antigen production. The purified bacterial lysate was utilized to detect mouse antibodies in the serological two-tiered test system (ELISA and western blot). Spirochetes were first cultured as described elsewhere [15]. When bacteria reached the late exponential phase (after five days), 150 μl were transferred into each of two 12-ml tubes (Centrifuge Tube 12; TPP, Faust Lab Science GmbH) containing 6 ml medium and were further incubated for three days. These 6-ml bacteria suspensions were transferred to a sterile glass bottle containing 1 l medium and incubated until late exponential phase of growth (five days of incubation). Antigen preparation was done via ultrasound disruption according to Töpfer et al. [24] and the centrifuged supernatant of the whole cell lysate was stored at -80°C until used. Determination of protein concentration and quality control of the antigen solution was carried out as described previously [24].

### Antibody detection with a kinetic ELISA

The microdilution plates (Nunc-Immuno Microwell Maxisorp C96; Thermo Scientific, VWR International GmbH, Ismaning, Germany) were coated with whole cell antigen lysate of *B*. *persica* at a concentration of 0.2 μg per well as described by Barth et al. [25]. Detection of specific antibodies against *B*. *persica* was done with a computer-assisted, kinetic-based ELISA after Shin et al. [26]. Serum and pooled plasma samples were diluted 1:100 in sample buffer containing PBS, 0.05% Tween 20 (neoLab Migge Laborbedarf-Vertriebs GmbH, Heidelberg, Germany) and 2% non-fat dry milk (Merck KGaA, Darmstadt, Germany). Four control serum samples were added in each run. Peroxidase-conjugated goat IgG fraction to mouse immunoglobulins (IgG, IgA, IgM; MP Biomedicals, LLC, Heidelberg, Germany) were diluted 1:4,000 in sample buffer and used as the secondary antibody. As a final step, substrate (TMB Microwell Peroxidase Substrate Kit; KPL, medac GmbH, Wedel, Germany) was added and after 1 min 45 s the extinction of each well was read five times in 35-s intervals at 650 nm in a SpectraMax Plus 384 Microplate Reader (Molecular Devices (UK) Ltd, Wokingham, United Kingdom). Results were calculated with the SoftMax Pro software 5.3 (Molecular Devices (UK) Ltd). To standardize the sample evaluation and for the comparability of the plates of each run, the results of the samples were adjusted to the evaluated values of the control samples. Graphs were constructed with the OriginPro 9.1 Software (Additive GmbH).

### Antibody visualization with western blots

For antigen preparation, three parts of antigen were mixed with one part of reducing sample buffer (Roti-Load 1; Carl Roth GmbH & Co. KG, Karlsruhe, Germany) and heated for 10 min at 90°C in a ThermoMixer comfort 5355 V 2.0 (Eppendorf Vertrieb Deutschland GmbH). The diluted antigen was loaded into a precast gel (4–15% Mini-PROTEAN TGX Stain-Free Precast Gels, IPG well comb, 86 x 67 mm (W x L); Bio-Rad Laboratories GmbH, Munich, Germany) and a protein weight ladder (Precision Plus Protein WesternC Standards; Bio-Rad Laboratories GmbH) was included separated from each other with a 5-mm wide polytetrafluoroethylene (PTFE) stick. Gel electrophoresis was performed with 1:10 diluted running buffer (10x Tris/Glycine/SDS Buffer; Bio-Rad Laboratories GmbH) in a Mini-PROTEAN Tetra cell (Bio-Rad Laboratories GmbH) at 250 V for 22 min. Western blot and immunodetection were done according to the Protein Blotting Guide (Bulletin #2895; Bio-Rad Laboratories GmbH) and a house-intern protocol as outlined below. Buffers and solutions were produced following recipes of the Protein Blotting Guide. Blotting of proteins onto a nitrocellulose membrane (MemBlot CN—Rolle, 0.45 μm, 10 x 7.5 cm; membraPure, Bodenheim, Germany) was carried out at 30 V for 960 min using a Mini Trans-Blot module (Bio-Rad Laboratories GmbH) in Towbin Buffer. The membrane was washed with tris-buffered saline (TBS, pH = 7.5) for 7 min and blocked for 1 h at room temperature in 5% non-fat milk-TBS. Subsequently, the membrane was washed with TTBS twice for 7 min (0.05% Tween 20 in TBS) and subsequently cut into strips (3–4 mm wide). Serum and plasma samples were diluted 1:100 in 5% non-fat milk-TTBS and incubated with the membrane strips for 1 h at room temperature hhhhhhh. Protein standard strips were incubated with plain 5% non-fat milk-TTBS. After washing (four times, 7 min, in TTBS), the strips were incubated with 1:1,000 diluted detection antibody in TTBS (peroxidase-conjugated goat IgG fraction to mouse immunoglobulins IgG, IgA, IgM; MP Biomedicals, LLC), and the strips with the protein standard were incubated with Streptactin solution (Precision Protein StrepTactin-HRP Conjugate; Bio-Rad Laboratories GmbH) for 1 h at room temperature, respectively. Strips were washed four times with TTBS for 7 min. After a final wash step with TBS for 1 min, color development was achieved by adding substrate (Opti-4CN Substrate Kit; Bio-Rad Laboratories GmbH) and stopped after 4 min by washing in distilled water. Images were taken with the CemiDoc MP System and Image Lab Software Version 5.0 (Bio-Rad Laboratories GmbH).

### Ethics statement

Mouse experiments were carried out according to the guidelines approved by the Animal Welfare Committee of the Sachgebiet 54, Regierung von Oberbayern (Munich, Germany). The animal care and use protocols adhere to the German Tierschutzgesetz, the Tierschutz-Versuchstierverordnung and the recommendations of GV-SOLAS.

## Results

### Kinetics of *B*. *persica* spirochetemia

**Fig 1:**
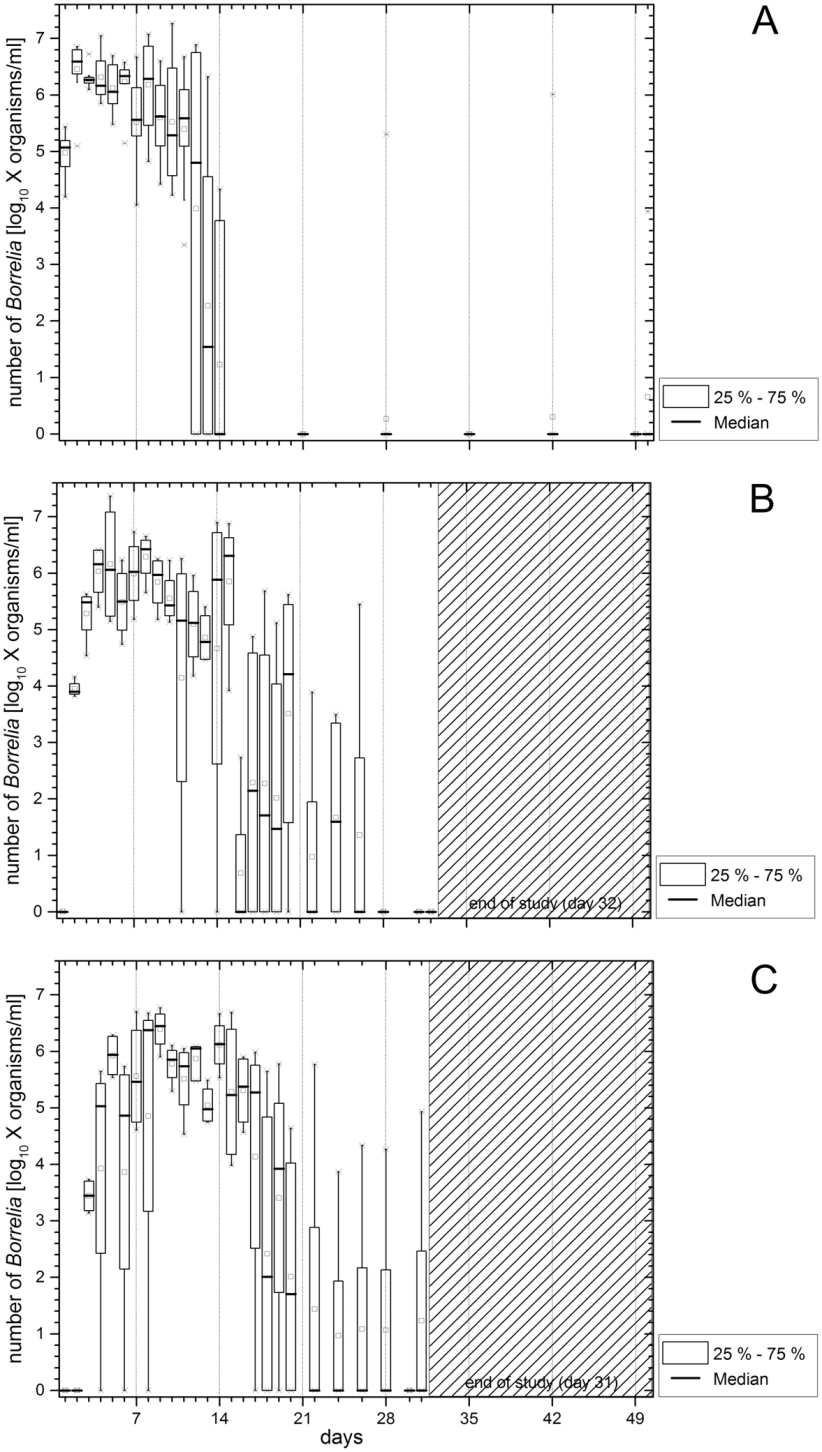
A-C. Spirochete burden in the blood. Results of real-time PCR detecting the *flaB* gene of *B*. *persica* in blood from C3H/HeOuJ mice that had received 1 x 10^6^ (Fig 1A), 1 x 10^4^ (Fig 1B) or 1 x 10^2^ (Fig 1C) *B*. *persica*/mouse. The absolute spirochete numbers in each blood sample (Y-axis, log_10_-scale) are plotted as box plots against the blood sampling days (X-axis).

For direct pathogen detection, DNA was extracted from murine blood samples and the *flaB* gene of *B*. *persica* was detected with a real-time PCR. Data are shown as box plots using a log_10_-scale of the absolute spirochete numbers per ml blood (Y-axis) and plotted against the blood sampling days (X-axis; Fig 1A–1C).

When mice were inoculated intradermally with 1 x 10^6^ *B*. *persica* organisms (Fig 1A), spirochetes were detectable in their blood starting one day after injection. Median spirochete concentration ranged from 4.80 to 6.59 (log_10_ x organisms/ml) during the first 12 days. A substantial decline in detectable spirochete numbers was observed from day 12 to 14. The median spirochete numbers dropped from 4.80 to 0 (log_10_ x organisms/ml). After day 14, the majority of the mice tested negative for spirochetes in the blood, while during the same period three mice showed reduced numbers of borrelia and only one of them produced a positive signal on the final day of the experiment. The highest spirochete burden observed in a blood sample of an individual mouse was 1.9 x 10^7^ *B*. *persica*/ml. When less spirochetes were used for intradermal inoculation (1 x 10^4^ and 1 x 10^2^ *B*. *persica*/mouse), spirochetes appeared in the blood circulation of the mice with a delay compared to the experiment performed with 1 x 10^6^ *B*. *persica*/mouse. When 1 x 10^4^ *B*. *persica* organisms were injected (Fig 1B), the earliest spirochetes were detectable two days after inoculation. Three peaks in spirochete concentration were recorded until day 15 (median spirochete concentration ranged from 3.90 to 6.43; log_10_ x organisms/ml) and then the spirochete number decreased from day 15 onwards (6.31 to 0; log_10_ x organisms/ml). After day 16, the median spirochete concentration varied at a low level (1.60 to 4.21; log_10_ x organisms/ml). The majority of the mice tested negative from day 26 onwards. The highest spirochete load observed in a blood sample of an individual mouse was 2.3 x 10^7^ organisms/ml. When 1 x 10^2^ *B*. *persica* organisms were injected (Fig 1C), spirochetes were detectable beginning on day 3. Varying spirochete numbers in blood were observed during the first 16 days of the experiment and from day 17 onwards the median number of spirochetes decreased substantially (from 5.28 to 2.01; log_10_ x organisms/ml). Mice showed spirochetemia at a low level until day 20. Then, the majority of the animals tested negative. No signals for *flaB* DNA were recorded for mice that were exposed to only four *B*. *persica* organisms per mouse and from mice which served as negative controls.

In summary, intradermal injection of decreasing numbers of *B*. *persica* resulted in delayed appearance of the spirochetes in the blood of infected mice. After an initial fluctuation of the median spirochete concentration at a high level, a sudden decrease in spirochete numbers was observed in each group from day 13 to 18. The infection rate was 100% when mice were injected intradermally with doses of 1 x 10^6^ (20/20), 1 x 10^4^ (8/8) and 1 x 10^2^ (8/8) *B*. *persica*/mouse. The lowest dose tested (four *B*. *persica*/mouse) did not result in infection of any mouse (0/8).

**Fig 2:**
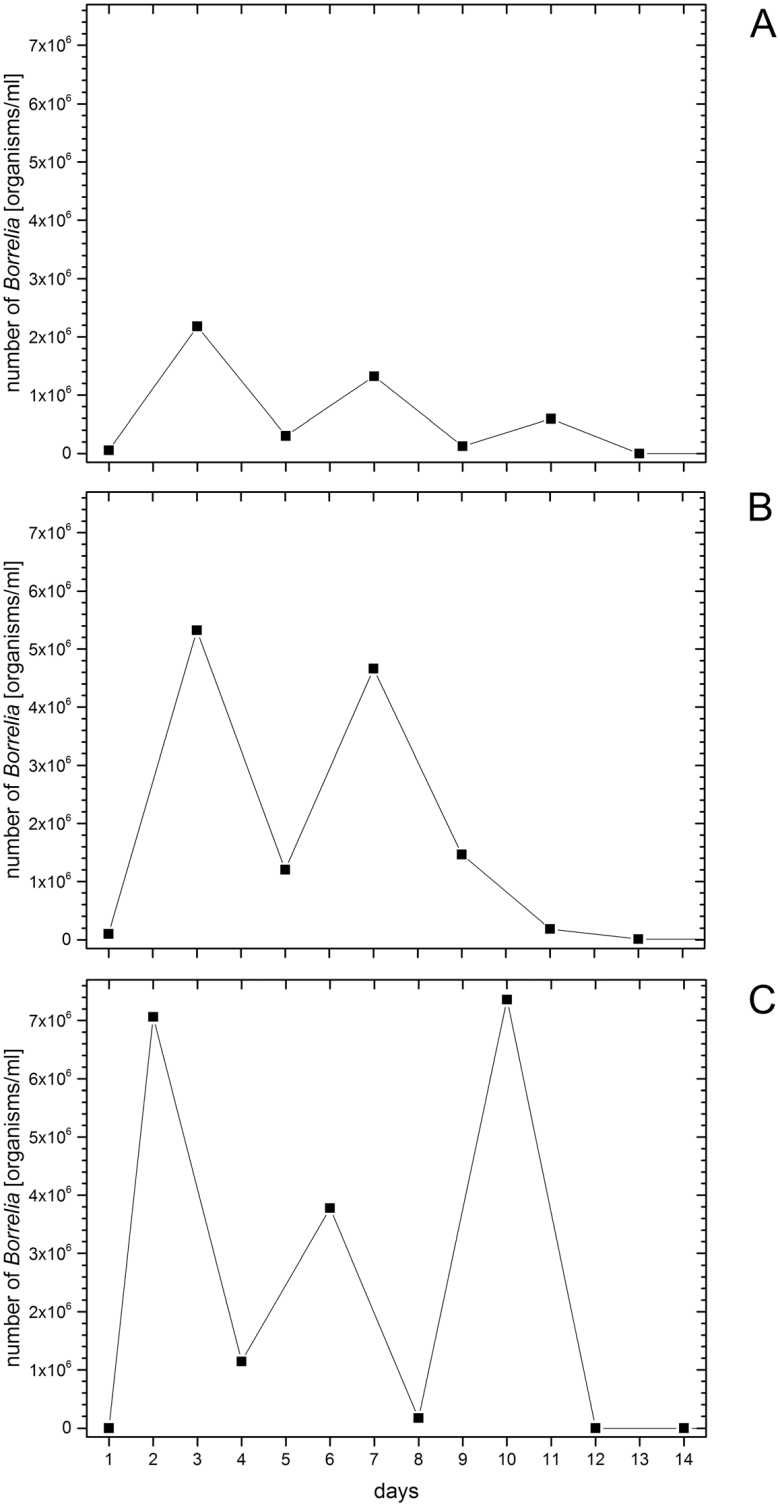
A-C. Individual relapse pattern. The absolute spirochete numbers per ml blood of three selected mice that had received 1 x 10^6^ *B*. *persica*/mouse (Y-axis) are plotted against the blood sampling days (X-axis). Mouse two (Fig 2A) and mouse 12 (Fig 2B) were part of subgroup one; mouse 18 (Fig 2C) was part of subgroup two. During the first 14 days, subgroups were bled according to an alternating sample collection schedule.

When analyzed at the individual level, most animals showed two to three peaks of spirochetemia and only a few (3/36) produced one peak. Fig 2A–2C show the absolute spirochete numbers per ml blood of three selected mice. These individual animals, which received 1 x 10^6^ *B*. *persica*/mouse, revealed three different relapse patterns. In terms of mouse #2 (Fig 2A), peak spirochetemia intensities declined over time. First, spirochete numbers increased up to 2.2 x 10^6^ organisms/ml on day 3. Subsequently, spirochetemia intensity decreased until day 5, rose up to 1.3 x 10^6^ organisms/ml on day 7, declined again on day 9 and showed a last small peak of 6.0 x 10^5^ organisms/ml on day 11. After that, the animal was negative until the end of the experiment. The lowest detectable number of spirochetes between the peaks was recorded on day 9 (1.2 x 10^5^ organisms/ml). In mouse #12 (Fig 2B), two peaks (5.3 x 10^6^ organisms/ml on day 3 and 4.7 x 10^6^ organisms/ml on day 7) were noted. Between the peaks the detectable spirochete concentration was 1.2 x 10^6^ organisms/ml. This mouse was negative after day 13. Fig 2C depicts the kinetics of spirochetemia in mouse #18: the first high peak with 7.1 x 10^6^ organisms/ml on day 2 was followed by a second low peak with 3.8 x 10^6^ organisms/ml on day 6, which again was followed by a last high peak with 7.4 x 10^6^ organisms/ml on day 10. Between the first and the second peak the lowest spirochete count was 1.1 x 10^6^ organisms/ml on day 4, while only 1.7 x 10^5^ organisms/ml were observed between the second and the third peak on day 8. This mouse also remained negative from day 12 onwards.

Interestingly, none of the infected mice showed any clinical signs or elevated temperatures during spirochetemia and the following periods when compared to negative control animals.

### Re-cultivation and real-time PCR detection of *B*. *persica* in tissues

Tissue samples were collected from the skin around the infection area, heart, spleen, urinary bladder, left tarsal joint, and brain at the end of the infection experiment with 1 x 10^6^ borrelia/mouse on days 49/50. Cultures with liquid medium were started to attempt the cultivation of the borrelia. The organ cultures were investigated for the presence of viable spirochetes under a dark-field microscope once a week over a period of three weeks. One week after initiating the cultures, at least some single borrelia organisms were observed in most brain cultures. After three weeks, 13 out of 20 observed brain samples were found positive with some cultures showing massive numbers of rapidly-moving spirochetes (Table 1). Three skin cultures were also observed as positive after three weeks. In total, 70% (14/20) of the infected mice tested positive by culture. By real-time PCR, 90% (18/20) of the infected mice tested positive for the *B*. *persica flaB* gene in tissue samples. In addition to 18 positive brain and three skin samples from 18 mice, one heart and one splenic sample of the same mouse were positive by real-time PCR (this mouse was also positive by blood real-time PCR performed on its final day of experiment). Control animals tested negative in both methods. According to the results, only brain and skin samples were investigated on the final days of the dose finding study. Spirochetes were seen in 87.5% (7/8; infection dose: 1 x 10^4^ *B*. *persica*/mouse) and 100% (8/8; infection dose: 1 x 10^2^ *B*. *persica*/mouse) of brain cultures. All tissue cultures of animals that had received four *B*. *persica*/mouse and all negative controls tested negative. Real-time PCR was 100% (8/8) positive for brain samples from mice infected with doses of 1 x 10^4^ and 1 x 10^2^ *B*. *persica*/mouse. None of the skin samples tested positive in both methods.

**Table 1:** Organ dissemination of *B*. *persica* following inoculation with different doses of spirochetes.

		Number of positive tissue samples and mice in culture/*flaB-*qPCR							
Infection dose	n	heart	spleen	bladder	joint	skin	brain	mice	mice in %
1 x 10^6^	20	0/1	0/1	0/0	0/0	3/3	13/18	14/18	70/90
1 x 10^4^	8	nd	nd	nd	nd	0/0	7/8	7/8	87.5/100
1 x 10^2^	8	nd	nd	nd	nd	0/0	8/8	8/8	100/100
4 x 10^0^	8	nd	nd	nd	nd	0/0	0/0	0/0	0/0

n, number of mice

nd, not done

### Histopathological findings

Five mice (inoculated with 1 x 10^6^ *B*. *persica*/mouse) which were positive according to all other test methods were selected for histopathologic evaluation. A negative mouse served as a control. The paraffin-embedded slices of brains and kidneys as well as the plastic-embedded slices of brains and joints revealed no histopathological changes indicative for inflammatory responses. Two specimens of in plastic-embedded kidneys (one infected and the uninfected mouse) contained small scattered interstitial infiltrations of lymphocytes (mild interstitial non-suppurative focal nephritis).

### Two-tiered test system (ELISA and western blot) for the detection of specific antibodies against *B*. *persica*

**Fig 3:**
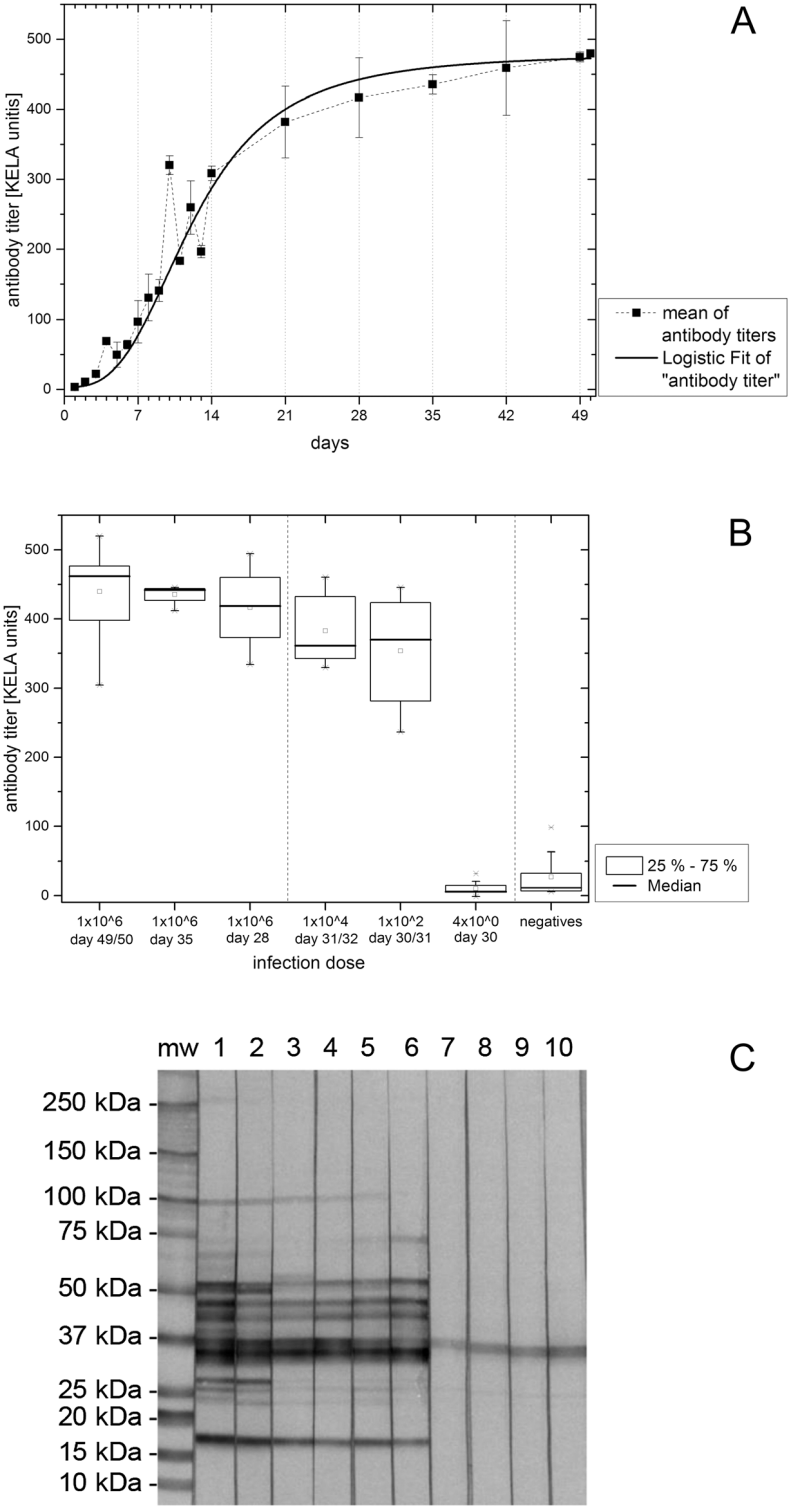
A-C. Antibody response against *B*. *persica*. Fig 3A. Antibody levels of pooled plasma samples (infection dose 1 x 10^6^ *B*. persica/mouse). The mean (calculated out of the values of the subgroup-specific pooled plasma samples) and the standard deviation (Y-axis) are plotted against the blood sampling days (X-axis). The logistic fitting is shown as a continuous line. Fig 3B. Antibody levels of all experimental infection dose groups are shown as box plots; serum samples from final days and pooled plasma samples from days 35 and 28. Fig 3C. Western blot of final sera from two randomly selected mice out of each infection dose group. Mw = molecular weight standard; 1, 2 = 1 x 10^6^ *B*. *persica*/mouse; 3, 4 = 1 x 10^4^ *B*. *persica*/mouse; 5, 6 = 1 x 10^2^ *B*. *persica*/mouse; 7, 8 = 4 x 10^0^ *B*. *persica*/mouse; 9, 10 = negative mice.

The specific antibody response against *B*. *persica* was measured with a kinetic ELISA and characterized by western blotting. Plasma samples of mice injected with 1 x 10^6^ *B*. *persica*/mouse were collected and pooled according to subgroups from day 1 to 50 according to an alternating sample collection schedule. Plasma samples as well as individual final serum samples of all animals were tested with an ELISA for the detection of murine IgM, IgG and IgA antibodies. Antibody levels developed immediately after spirochete injection and rose to 381.6 KELA units until day 21. Antibody levels plateaued (416.4 to 479.5 KELA units) until day 50 (Fig 3A). Antibody levels of individual final serum samples are shown in Fig 3B. The highest antibody levels were obtained in animals injected with 1 x 10^6^ *B*. *persica*/mouse on days 49/50 of the experiment. Mice exposed to 1 x 10^4^ or 1 x 10^2^ *B*. *persica*/mouse showed medium to high levels of specific antibodies, however their antibody levels were lower when compared to the high-dose exposed group on day 28 (pooled plasma samples). Sera of animals receiving only four *B*. *persica*/mouse and negative mice showed non-specific antibody responses.

Western blots of individual final sera from each infection dose group showed bands between 15 kDa and 100 kDa. The patterns of the infected mice were similar. Nevertheless, the lower antibody levels induced by the smaller infection doses were reflected in the intensities of the immunoblot bands. Negative controls and animals that had received four *B*. *persica*/mouse showed only non-specific bands at 25 kDa and 37 kDa (Fig 3C).

## Discussion

A murine infection model for *B*. *persica* strain LMU-C01 was established in this study. Investigated parameters such as clinical signs, spirochete burden, target organs of infection, histopathology and antibody response should provide further insights into the development of TBRF. Intradermal infection was 100% successful for the infection doses of 1 x 10^6^, 1 x 10^4^ and 1 x 10^2^ spirochetes per mouse. The dose of four *B*. *persica* organisms per mouse did not initiate infection in any mouse. This seems contradictory to other studies in other RF species, in which infection succeeded with single spirochetes of *B*. *recurrentis* var. *turicatae* [27] and *B*. *hermsii* [28]. It seems as though the pathogenicity of different RF species or even isolated single strains is decisive for the minimal infection dose and for a successful infection in animals. Furthermore, the route of infection might be a crucial factor for the initiation of an infection. Whereas in other studies animals were infected intraperitoneally with different RF species [27, 28], mice were infected intradermally with *B*. *persica* in our study. It is possible that innate defense mechanisms involving the skin immune cells might kill off a certain number of injected spirochetes. This could be a reasonable explanation for the unsuccessful intradermal infection with the infection dose of only four *B*. *persica* per mouse. To our knowledge, the number of transmitted *B*. *persica* spirochetes by its vector *Ornithodoros tholozani* during the feeding process is unknown. However, one can speculate that the number of transmitted spirochetes is low, because of the short (minutes) blood meal of soft ticks [29] compared to hard ticks (several days of attachment on the host; [30]). Up to 1 x 10^4^ *B*. *burgdorferi* organisms have been found in infected *Ixodes scapularis* (formerly *Ixodes dammini*) nymphal ticks after feeding on experimentally infected mice [31]. And yet it is unknown how many *B*. *burgdorferi* spirochetes are transmitted exactly during the feeding process from *Ixodes scapularis* to the host animal.

Spirochete burden in the blood of the infected animals was dependent on time and infection dose, and varied within the study groups displaying individual kinetics and extent of spirochetemia. High doses of inoculated *B*. *persica* induced a prompt appearance and an early high load of *B*. *persica* in the circulation, but had just a small influence on the maximal spirochete number. At the same time, these early large numbers of bacteria seem to have effectively stimulated the mice’s immune response. Most of the mice in the group that had received 1 x 10^6^ spirochetes per mouse had cleared spirochetemia by day 14, while the other two groups (1 x 10^4^ and 1 x 10^2^) controlled their spirochetemia days later or incompletely (compare Fig 1A, 1B and 1C). During days of investigation mice showed individual relapse patterns with varying numbers of bacteria (Fig 2A–2C). Such fluctuations in spirochete presence of individual mice were also seen in a study in Jordan [20]. In our study, every infected mouse showed one to three peaks of spirochetemia during the time course of blood sampling. Spirochetal numbers were low between the peaks, but still detectable by real-time PCR. To our knowledge, previous publications did not investigate the genetic mechanisms of the cyclic nature of *B*. *persica* at the level of variable major proteins (VMPs) as described for other RF borreliae [32]. However, VMP sequences have been identified for *B*. *persica* (e.g. variable large protein 18 under NCBI accession number: WP_024653159). Changes in VMPs expression are likely to be associated with recurring spirochetemia presented in this study. Further investigations are required to explore the VMPs and the underlying gene sequences for *B*. *persica* in more detail. The infection of our mice resulted in similar maximal bacteria numbers per ml when compared to an earlier study in guinea pigs (~6.8 x 10^6^ *B*. *persica*/ml [13]) and so far, these animals have routinely been used to study and maintain *B*. *persica* [12–14]. Interestingly, the mouse strain used in this study (C3H/HeOuJ) did not show any clinical signs of disease. Other relapsing fever spirochetes were reported to have varying influences on temperature profiles in mice. For example, *B*. *microtti* induced fever in white mice (mouse strain unknown; [14]). Nevertheless, our infection model could be useful for research, because it allows comparative studies with other RF spirochetes in mice [16–19].

An interesting and crucial aspect of RF infections is the target tissues/organs, since these locations of spirochete persistence determine the final outcome of infection. Data obtained in this study clearly show that *B*. *persica* disseminates into the brains of mice (Table 1). Babudieri reported already in 1957 [20] that uncharacterized spirochete isolates from Jordan survived in mice’s brains after experimental infection. Similarly, *B*. *crocidurae* and *B*. *duttoni* are known to infiltrate the brains of mice [18]. In humans, neurological symptoms due to *B*. *persica* infection are rarely reported (reviewed in [33]). It is, therefore, not surprising that the mice in this study did not display clinical signs of brain infection. Results of our histopathological investigations also support the assumption that short-term infections with *B*. *persica* not necessarily lead to apparent clinical signs. Whether the latent brain infection in immunocompetent mice remains without any inflammatory response needs to be evaluated. Further studies aiming at long-term infections or at reactivation of the spirochetes as a result of immunosuppression could shed some light on this issue. Spirochetes were also detected in skin samples from five mice which received an inoculum of 1 x 10^6^ organisms (two mice positive in culture, two mice positive for specific DNA, one mouse positive for both). Furthermore, one heart tissue sample and one splenic sample taken from the same mouse (1 x 10^6^ *B*. *persica*/mouse) tested positive for *B*. *persica flaB* gene. Interestingly, the blood sample of this mouse collected on the final day of experiment (day 50) was also positive for *B*. *persica* DNA. Since the heart and the spleen are blood-rich organs, it is likely that these positive results indicate the presence of *B*. *persica* in the circulation rather than dissemination to these organs. The facts that the blood-rich organs of the other animals (infection dose 1 x 10^6^ *B*. *persica*/mouse) were negative and that no borrelial DNA was detectable in the blood samples during the final days of the experiments suggest that brain and skin infections in these animals were real rather than due to contamination with spirochetemic blood.

Our histopathological investigations revealed mild interstitial non-suppurative focal nephritis in two kidneys from an infected and an uninfected mouse. It seems that the findings in our mice are an incidental event considering the changes in the kidney of the negative control animal. It is necessary to focus further investigations on this *B*. *persica* strain to clarify the non-conclusive histopathological results we had seen using a larger number of tissue samples or examining organs earlier post infection.

Comparing all tests used in this study, the highest detection rate for infection was achieved by antibody detection followed by real-time PCR performed on repeated blood samples collected during the first week of infection. Results of real-time PCRs performed on tissue samples (brains) also produced high detection rates. Yet, tissue cultivation is the most difficult and error prone method due to contamination with other fast-growing bacteria.

With the infection model presented here, further investigations are possible in order to gain advanced insights into the pathogenesis of *B*. *persica* infection and to characterize the host immune response mounted against in detail. We propose that this murine model could also be useful for the development of further diagnostic methods for treatment studies in order to detect, clear or prevent the infection with *B*. *persica*.

### Conclusion

The results of this study show that *B*. *persica* strain LMU-C01 can be used to establish infection in immunocompetent C3H/HeOuJ mice. The minimal infectious dose was between four and 1 x 10^2^ *B*. *persica* organisms by intradermal inoculation in this study. Spirochetes were detected in the blood, brain and skin tissue samples thereby defining the brain and the skin as target organs of *B*. *persica* dissemination. The infection model presented in this study can serve as a platform for further ensuing in vivo investigations to gain new insights into the pathogenesis of *B*. *persica*.
